# Comprehensive Behavioral Intervention for Tics reduces perception-action binding during inhibitory control in Gilles de la Tourette syndrome

**DOI:** 10.1038/s41598-020-58269-z

**Published:** 2020-01-24

**Authors:** Vanessa Petruo, Benjamin Bodmer, Annet Bluschke, Alexander Münchau, Veit Roessner, Christian Beste

**Affiliations:** 1 Cognitive Neurophysiology, Department of Child and Adolescent Psychiatry, Faculty of Medicine, TU Dresden, Germany; 2 Department of Pediatric and Adult Movement Disorders and Neuropsychiatry, Institute of Neurogenetics, Center for Brain, Behavior and Metabolism, University of Lübeck, Lübeck, Germany

**Keywords:** Cognitive control, Human behaviour

## Abstract

Gilles de la Tourette Syndrome (GTS) is a developmental disorder. Empirical studies and an emerging cognitive framework on GTS suggest that GTS is a disorder of abnormally strong ‘perception-action binding’. Theoretical considerations imply that the effectiveness of long-established behavioral interventions might be related to a normalization of increased binding in GTS. This has not been tested yet. We examined the effect of a standardized Comprehensive Behavior Intervention for Tics (CBIT) in N = 21 adolescent GTS patients and N = 21 healthy controls on perception-action binding in an inhibitory control paradigm. Prior to CBIT, GTS patients showed compromised performance compared to controls, specifically when inhibitory control was triggered by uni-modal visual compared to bi-modal stimuli. After CBIT intervention, GTS patient’s performance was at the same level as healthy controls. This is supported by a Bayesian data analysis. CBIT specifically affected inhibitory control in a condition where reconfigurations of perception-action bindings are necessary to perform inhibitory control. A power of 95% was evident for these effects. CBIT reduces increased ‘binding’ between perception and action in GTS and thereby increases the ability to perform response inhibition. The results are the first to provide insights as to why CBIT is effective by relating elements of this intervention to overarching cognitive theoretical frameworks on perception-action bindings.

## Introduction

Gilles de la Tourette Syndrome (GTS) is a developmental disorder with multi-faceted neuropsychiatric symptoms, as onset and highest prevalence in childhood or adolescence and is characterized by multiple motor and vocal tics^1,2^. Traditionally, GTS has been considered as a movement disorder. This is not undisputed though given that most tics can, at least partially, be controlled, are associated with premonitory sensations and might reflect motor learning and habit formation^3^. On the basis of these findings, it has recently been suggested that a hallmark of GTS is an abnormally strong interrelation of perceptual processes and motor actions, particularly between premonitory sensation including preceding urges and tics, and that GTS might be conceptualized as a disorder in which purposeful actions play an important role^3^. Indeed, several studies have reported that GTS patients make better use of multi- or bi-modal sensory stimuli for response selection^4,5^. Moreover, findings from procedural learning also suggest that GTS patients establish connections between stimuli and the corresponding response faster and more strongly^6,7^. In line with this, GTS is associated with increased habit formation tendencies^8^ depending on the establishment of strong stimulus-response mappings. These findings corroborate the notion that GTS might be a disorder characterized by an abnormally strong ‘binding’ between perception and action, for which a cognitive framework based on the Theory of Event Coding (TEC) detailing the link between perception and action is an attractive overarching concept for GTS^3,9^. The TEC framework states that whenever a stimulus presented and triggers a response, stimulus-action bindings are established. These bindings (associations) are stored in ‘event files’^10^. Once such bindings have been established, they affect subsequent actions, particularly when these are executed on the basis of only a slightly altered stimulus input. In such cases, previously established and still existing stimulus-response associations/bindings impede behavioral control^10–12^, because expectations about stimulus-response mapping become violated. Therefore, the *context* established by previous stimulus-response mappings profoundly affects a to be executed action. In a previous study by our group^13^, we provided evidence that a stronger contextual representation of stimulus-response bindings in GTS patients impedes performance to inhibit prepotent responses, when the same inhibitory control processes had to be exerted upon different sensory input.

The most established behavioral intervention technique aimed at reducing tics in GTS is habit reversal training (HRT)^14–16^. Data have already provided evidence for the efficacy of HRT in reducing tics in GTS^17,18^. HRT is based on the concept that patients learn to perceive premonitory urges before the tic and to apply antagonistic, competing muscle activity (movement or tension) to inhibit or counteract the occurrence of the tic. HRT builds the basis for the Comprehensive Behavioral Intervention for Tics (CBIT), for which studies also revealed a high efficacy to treat tics^19,20^. In CBIT, different psychoeducational elements, relaxation training and reward contingency plans are added to the HRT methods. According to an emerging view, tics may represent prefabricated actions stored in event files, which can be triggered by the appropriate perceptual input^3^. Replacing a tic by another response, as trained by the HRT component in CBIT, may therefore train to restructure or unbind tic-specific event files. It is possible that CBIT trains not only the tic-specific, but also the general ability of GTS patients to restructure event files. From a cognitive-theoretical, including the TEC perspective on GTS, the HRT component in CBIT may be effective because it restructures tic-specific event files. If this is the case, and the relevant mechanism underlying the HRT component in CBIT can be framed as a restructuring of event files, it is possible that the CBIT does also affect perception-action bindings impeding the ability to inhibit prepotent responses, when the same inhibitory control processes has to be exerted following different sensory input^13^. In the current study, we test the hypothesis that a standard CBIT intervention reduces such contextual modulations of stimulus-response bindings in adolescent GTS patients. To test this hypothesis we performed a follow-up behavioral study of the adolescent GTS patients investigated previously^13^. We examined performance in a response inhibition task (Go/Nogo task) and focused on the frequency of erroneous response executions in Nogo trials (i.e. false alarm rates). The Go/Nogo task was designed in a way that bindings between stimuli and responses facilitate the execution of erroneous responses in one condition of the task. In that particular condition the stimulus input was altered, as compared to the other two Nogo conditions and therefore caused problem in response inhibition whenever there is a strong binding between the more common combination of stimuli and responses (response inhibitions) in the other conditions of the task.

## Results

For the reaction times (RTs) in the Go conditions, the ANOVA only revealed a main effect “condition” (F(1,40) = 35.94; p < 0.001; η_p_^2^ = 0.473), showing that the reaction times were faster in the Go_compatible_ condition (496 ± 13 ms), than in the Go_without_ condition (521 ± 11 ms). No other main or interaction effects were significant (all F <1.6; p > 0.2).

For the accuracy on Go trials, the ANOVA revealed no significant main or interaction effect (all F < 1.3; p > 0.2) and the overall rate of correct responses on Go trials was 95.71% (±2.1). For the rate of missed response on Go trials, the ANOVA only revealed a main effect “time point” (F(1,40) = 4.17; p = 0.048; η_p_^2^ = 0.094), revealing that the rate of missed responses on Go trials was smaller at time point T2 (1.89 ± 0.61) than at T1 (2.92 ± 0.99). No other effects were significant (all F <1.05; p > 0.3).

**Figure 1:**
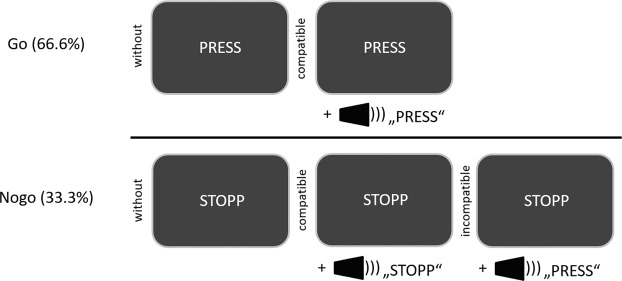
Data on the rate false alarms (responses in NoGo trials) is shown for time point T1 (left) and time point T2 (right). The x-axis denotes the different NoGo conditions in the task. GTS patients are shown in black, healthy controls in grey. The means and SEMs are given. *p < 0.05; ***p < 0.001.

However, the most important behavioral parameter is the rate of false alarms (FAs). For FAs, a main effect “condition” (F(2,80) = 76.89; p < 0.001; η_p_^2^ = 0.658) showed that FAs were highest in the NoGo_incompatible_ condition (25.59 ± 2.5), followed by the NoGo_without_ condition (18.19 ± 1.5) and the NoGo_compatible_ condition (10.76 ± 1.6). All conditions differed from each other (p < 0.001). Importantly, there was an interaction “time point × condition × group” (F(2,80) = 4.90; p < 0.01; η_p_^2^ = 0.109), indicating that the false alarm rate differed depending on whether GTS patients or controls were tested and whether testing took place before or after the treatment. The post-hoc power calculations revealed a power greater than 95% and it is shown that the effect size of this interaction is larger than that shown to be detectable in the sensitivity analysis (see methods section). No other main or interaction effect was significant (all F <1.2; p > 0.3). The interaction “time point × condition × group” is shown in Fig. 1.

A detailed analysis of this interaction using ANOVAs revealed the following for each time point separately: As can be seen in Fig. 1, there seem to be differential effects between experimental conditions and groups at time point T1, but not at T2. This is also revealed by the statistical models: For time point T1, the ANOVA revealed a main effect “condition” (F(2,40) = 55.46; p < 0.001; η_p_^2^ = 0.581), showing that FAs were highest in the NoGo_without_ condition (28.11 ± 3.1), followed by the NoGo_incompatible_ condition (20.04 ± 2.1) and the NoGo_compatible_ condition (8.83 ± 1.4). All conditions differed from each other (p < 0.001). Importantly, and in line with the previous publication using a larger sample size of GTS and HCs^13^, there was an interaction “condition × group” (F(2,40) = 3.58; p = 0.032; η_p_^2^ = 0.082). It is shown that in the NoGo_without_ condition (t(40) = −1.99; p = 0.015), the GTS group performed worse than the HCs group. No difference was evident in the NoGo_incompatible_ condition and the NoGo_compatible_ condition (all t < 1.01; p > 0.3). This is completely in line with the previous results by Petruo *et al*. (2018) and cross-validates these results. At time point T2, the ANOVA revealed a main effect “condition” (F(2,40) = 53.40; p < 0.001; η_p_^2^ = 0.572), showing that the FAs were, again, highest in the NoGo_without_ condition (24.28 ± 3.1), followed by the NoGo_incompatible_ condition (16.81 ± 1.8) and the NoGo_compatible_ condition (6.66 ± 1.2). All conditions differed from each other (p < 0.001). Importantly, there was no interaction “condition × group” anymore (F(2,40) = 0.11; p = 0.950; η_p_^2^ = 0.001). This is corroborated by a Bayesian data analysis using the method of Masson (2011), in which the probability of the null hypothesis being true given the obtained data *p*(H_0_/D) can be calculated. For the interaction effects “condition × group” at time point T2, this Bayes statistic revealed *p*(H_0_/D) = 0.97 and thus very strong evidence in favor for the null hypothesis. This suggests that after the CBIT intervention, no differential effects between GTS patients and controls were evident. Further tests showed that FAs in the NoGo_without_ condition decreased from time point T1 to T2 in the GTS group (t(20) = 4.25; p < 0.001). No changes in FAs between testing time points were evident in the healthy control group (t(20) = −0.15; p > 0.8). This shows that the interaction effect is driven by CBIT-induced changes in the GTS group in the NoGo_without_ condition. Correlation analyses showed that there was no linear relationship between the reduction of tics/symptoms between T1 and T2 in the GTS group (as examined using the YGTSS score) and the observed changes in the NoGo_without_ condition in GTS patients (r < 0.23; p > 0.4).

For the reaction times in erroneous NoGo trials, the ANOVA revealed no significant main or interaction effect (all F < 1.1; p > 0.3).

Importantly, the pattern of results for the false alarm rate data was not biased by comorbidities of the patients, or medication status. When including an additional between subject factor “comorbidity” (“yes or no”) as well as “medication” (“yes” or “no”) in the ANOVA, the results were not affected (all F < 0.55, p > 0.45). In further analyses we examined whether the scores at time point T1 in the M.I.N.I. KID and CY-BOCS modulated the pattern of results. To this end, we included these scores as a covariate in the statistical models. It is shown that these covariates did not have a significant effect (all F < 0.76, p > 0.33) and also the pattern of the other effects was unaffected. All these analyses show that the effects obtained are robust.

## Discussion

In the current study, we tested the effects of CBIT in children and adolescents with GTS patients on perception-action binding that was previously tested in these patients in a cognitive neurophysiological study^13^. The current findings significantly extend these prior data by showing how a well-established and clinically relevant intervention to reduce tics in GTS affects perception-action bindings. Doing so, the current study connects a standard clinical intervention to a new and emerging concept of GTS, i.e. GTS and its symptoms may reflect an abnormally strong ‘binding’ between perception and action^3^. These aspects cannot be inferred on the basis of previously published data^13^ using the paradigm that is also applied in the current study. CBIT has been shown to reduce tics in GTS^17,18^, and this was also shown in the current study (cf. reduction in YGTSS scores). We hypothesized that a CBIT intervention would reduce, i.e. normalize, increased perception-action bindings in these patients, which has previously been shown to impede the ability to inhibit prepotent responses when the same inhibitory control processes had to be exerted upon different sensory input^13^. The obtained behavioral data support this hypothesis.

The false alarm rate data reflecting the tendency to execute a response in NoGo trials (refer Fig. 1) did not differ between GTS patients and HCs in any of the experimental conditions of the Go/Nogo task. This was supported by Bayesian analysis providing very strong evidence for the null hypothesis. GTS patients and HCs did not differ from each other in the NoGo_compatible_ and the NoGo_incompatible_ condition prior to the conduction of CBIT. In the NoGo_compatible_ and the NoGo_incompatible_ condition, concurrent auditory information was evident that was either facilitating or impeding performance to inhibit a response in these NoGo trials. In the NoGo_compatible_ and the NoGo_incompatible_ conditions, both groups revealed modulatory effects that are well in line with the literature^21,22^, i.e., worse response inhibition performance when auditory information was conflicting with behaviorally relevant visual information. This pattern was unchanged following CBIT.

In contrast, performance in the NoGo_without_ condition differed between groups at baseline (i.e. prior the CBIT in the GTS group). Since the NoGo_without_ condition occurred as often as the multi-modal NoGo conditions (i.e. frequency NoGo_without_ = frequency NoGo_incompatible_ + frequency NoGo_compatible_), the group difference in the NoGo_without_ condition cannot be attributed to a simple trial frequency effect. The important characteristic of the NoGo_without_ condition is that responses have to be inhibited on the basis of purely visual information. In the other conditions, concurrent auditory information was evident. Thus, in the NoGo_without_ condition, action control has to be exerted on the basis of altered/reduced perceptual information; i.e. in an altered context. Whereas before the intervention, the false alarm rate was significantly larger in GTS patients indicating deficits in adapting performance to an altered context, there was no group difference following the CBIT anymore, i.e. false alarms were reduced by the CBIT intervention in GTS patients. Thus, CBIT normalizes or at least reduces the increased ‘binding’ between perception and action in GTS and thereby increases the ability to perform response inhibition. The results that CBIT normalized perception-action bindings in GTS patients to the level of HCs is supported by the Bayesian data analysis results. The finding that the control group did not reveal modulations in performance in the NoGo_without_ condition between the repeated testings clearly shows that the changes in the data pattern are driven by the CBIT intervention in the GTS group. However, the statistical analysis showed that even though there was a reduction of tics, as assessed using the YGTSS, there was no direct linear correlation between the changes in the task performance and the reduction of tics in the YGTSS. Although this might at first sight undermine the concept and interpretation that the clinical symptoms in GTS and perception-action share a common ground it is important to consider that correlations can only capture linear relationships. However, whether the relationship between tics/GTS symptoms and the degree of alteration in perception-action bindings is in fact linear, is currently unclear. Moreover, it is important to consider that the YGTSS, albeit very useful to capture the overall severity of GTS, is not an objective tic measure because it is based on patient interview or and clinical judgement and taking into account aspects other than tic frequency including symptom intensity, distribution, complexity and interference with other activities. A more objective clinical measures (like the Rush Score^23^) might have been better suited to directly capture tic frequency. This, however, was not available in the current study, which is a limitation. However, the obtained findings can be explained as follows:

According to the TEC framework, deficits in performance may occur when identical action programs have to be executed on the basis of altered stimulus input^10^. This is particularly the case when bindings between perception and action, i.e. between stimulus and response features within an event file are particularly strong^10,11,24^. Bi-modal stimuli establish strong stimulus-response associations^25^, particularly in GTS^5^. It has been suggested that difficulties to reconfigure an event file and to bind uni-modal stimuli to the same action (i.e. the inhibition of a response) is difficult in GTS^13^. Since behavioral performance in the uni-modal NoGo_without_ condition was improved by the CBIT intervention, the data suggest that the CBIT fosters the ability to reconfigure event files in GTS. It seems that as a consequence of the CBIT intervention GTS patients are better able to reconfigure event files and perform the same action (i.e. the inhibition of a response) on the basis of an altered sensory input. Behavioral interventions like HRT as the basis of CBIT aim at modifying the association between tics and triggering factors and tics^19,20^. During HRT/CBIT, patients learn to perform an action that is physically incompatible with the tic^20,26^; i.e. they learn to associate identical antecedent conditions or sensory information (e.g. pre-monitory urges) with different actions. From a cognitive-theoretical, or a theory of event coding perspective, the HRT/CBIT modulates factors playing an important role in perception-action bindings and patients learn to better reconfigure event files leading to tics. In the current study, high levels of cognitive control in the NoGo_without_ condition require to perform similar actions on the basis of altered sensory input. While this is not directly trained in HRT/CBIT, the important aspect is that in both cases (i.e. ability to associate different actions with the identical sensory information or the different sensory input to similar actions) a reconfiguration of event files is necessary. From a TEC perspective on GTS^3^, it seems that HRT/CBIT trains not only the tic-specific, but also the general ability of GTS patients to restructure event files. Therefore, HRT/CBIT is not only effective to reduce tics^19,20^, but also normalizes alterations in higher level cognitive functions. While this has also been shown previously^27^, the results from the current experiment directly testing effects of bindings between perception and action are the first to provide insights into the mechanisms in relation to influencial cognitive theoretical frameworks that can explain why HRT/CBIT is effective. However, the current findings cannot answer the question whether the HRT/CBIT actually reduces abnormally strong bindings between perception and action in GTS that may emerge as a consequence of structural and functional changes in fronto-striatal circuits of^3,28,29^, or does ‘just’ increase the patient’s ability to handle abnormally strong bindings between perception and action more efficiently. An important aspect to consider is whether the effects obtained simply reflect learning effects in the sense that the task becomes easier to accomplish and that differences in learning effects in GTS and controls may contribute to the pattern of results. Even though this cannot be fully excluded due to the limitation of the study that no GTS group was included that did not receive any intervention, it is unlikely that the effects are simply due to learning or task-familiarization effects. The reason is that learning or task-familiarization leads to more automated processes^30^. Importantly, response inhibition processes are compromised by more automated responding^31–36^. If the observed effects had come about through pure learning, this would have led to a higher degree of automation in the reaction tendency. As a consequence, the rate of false alarms would have increased. This, however, was not the case in any of the conditions in the applied paradigm. Therefore, learning effects are unlikely to explain the pattern of results.

## Materials and Methods

### GTS patients and healthy controls

This is a follow-up study of Petruo *et al*. (2018) testing patients with GTS diagnosed according to DSM-IV using an auditory-visual Go/NoGo task. For the follow-up, a subsample of N = 21 GTS out of N = 35 previously studied patients between 9 to 19 years was available (mean age 13.09 ± 2.1; mean IQ = 107.13 ± 10.5). These N = 21 patients were tested at baseline and 8 weeks later following CBIT training that was carried out in the outpatient clinical of the Department of Child and Adolescent Psychiatry (Faculty of Medicine, TU Dresden, Germany). The Yale Global Tic Severity Scale (YGTSS) was administered before and after the conduction of the CBIT protocol. Only this scale was assessed before and after the CBIT training, since the training is specifically targeted to reduce tics. CBIT reduced tics as indicated by a significant decrease in the YGTSS score between the time point before CBIT training (39.85 ± 2.81) and after CBIT training (29.41 ± 3.51) (t(20) = 4.89; p < 0.001). The mean total TS-score before CBIT intervention was 17.38 ± 2.12 and decreased to 13.04 ± 1.85 after CBIT intervention (t(20) = 4.01; p < 0.001). Before CBIT intervention clinical scales such as the, M.I.N.I. KID (Mini International Neuropsychiatric Interview for Children and Adolescents), CY-BOCS (Children’s Yale-Brown Obsessive-Compulsive Scale), were conducted, but not after the CBIT training, since CBIT training mainly target tics. Out of the N = 21 patients, N = 1 also had a diagnosis of attention deficit hyperactivity disorder and N = 5 a diagnosis of obsessive-compulsive disorder with a mean CY-BOCS Score of 8.5 (±3.11). N = 4 of the patients were on medication during both time points including treatment with Tiapride (N = 2), Aripiprazole (N = 1), or Fluoxetine (N = 1). The doses of the drugs were not changed between the testing time points T1 and T2.

A sample of N = 21 age and gender-matched healthy controls (HCs) was also tested twice. The HCs did not show psychiatric disorders as indicated by the M.I.N.I. KID and had no history of psychiatric disorders. The mean age was mean age 12.78 ± 2.3 years, the mean IQ was 105.78 ± 9.8. The time delay between the testing was the same as for the GTS group. As with the previous study, the groups did not differ regarding the distribution of males and females (chi^2^ < 0.024, p = 0.90) and there were no age or IQ differences (t < 0.2; p > 0.7).

### Power considerations

For the available sample size for the GTS and the control group, a ‘sensitivity analysis‘ was conducted using G*Power^37^. This analysis shows that with the used study design an effect size of f = 0.22, which equals a partial eta squared (η_p_2) of 0.05, can be detected with a power of 90% (5% type 1 error probability). Notably, the obtained effect size in the critical effect was larger (i.e. η_p_^2^ = 0.109; see results section). This shows that the study is sufficiently powered to reveal reliable effects.

### Ethical considerations

The study was approved by the ethics committees of the Universities of Dresden and Lübeck. Written informed consent was obtained from the subjects and their legal guardians. All methods and study procedures were performed in accordance with relevant guidelines and regulations.

### Task

**Figure 2:**
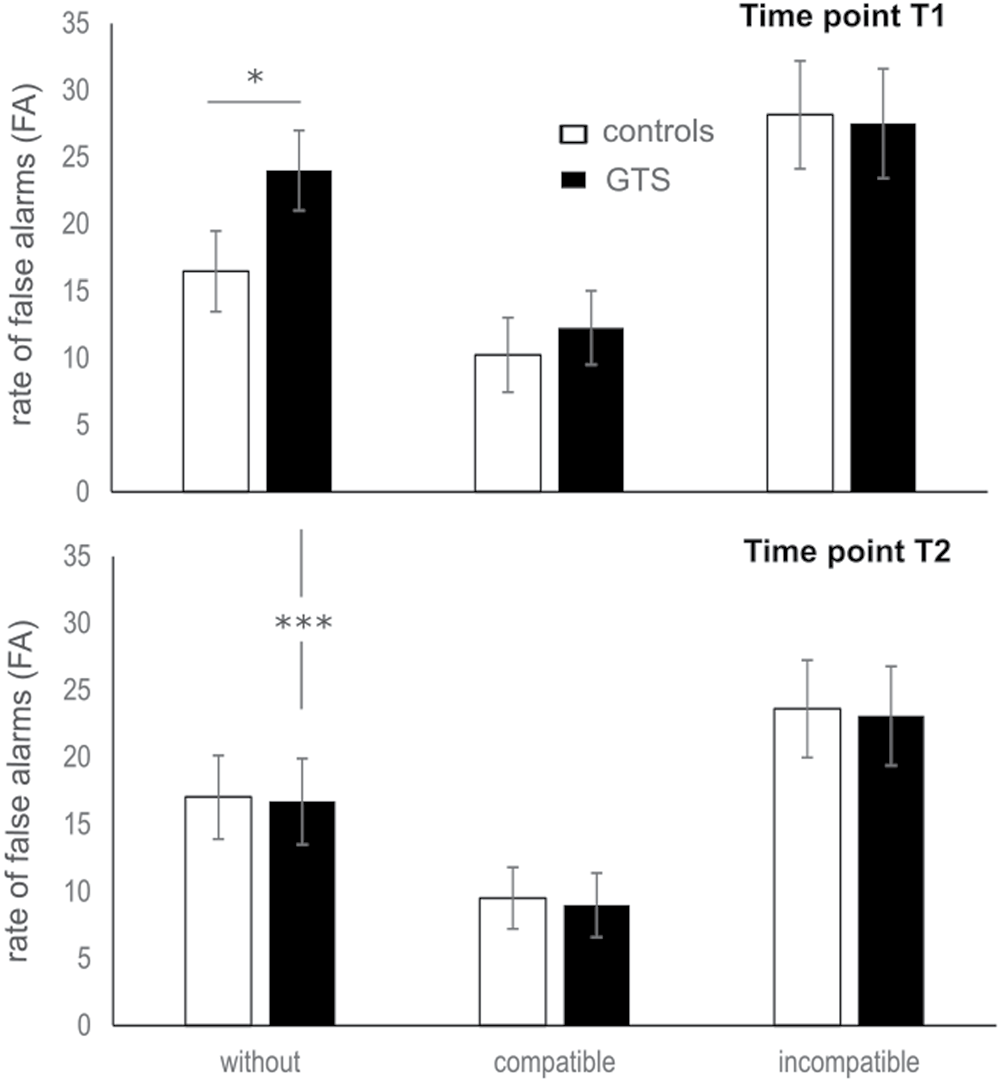
Illustration of the experimental paradigm. Upon presentation of the “PRESS” stimulus, a response had to be executed. Upon presentation of the “STOPP” stimulus, the response had to be inhibited. These conditions could either occur without or with concomitant auditory stimuli, or with an auditory stimulus. The experimental setup creates a context in which trials with bi-modal stimuli were more frequent than trials with uni-modal stimuli. Therefore, this context can interfere with performance in the Nogo_without_ condition.

The task, an auditory-visual Go/NoGo task, was identical to a previous study in GTS patients^13^. It is shown in Fig. 2.

During NoGo trials, the word stop (German: STOPP) was presented on the screen and participants had to refrain from responding. During Nogo trials, auditory sensory information was presented simultaneously to the visual NoGo stimulus. The stimuli were spoken words “DRÜCK” or “STOPP” (spoken by the neutral female computer voice of “google translate”). The length of visual and auditory stimuli was the same (400 ms). There were N = 72 NoGo trials in which an auditory NoGo stimulus (“STOPP”) was presented. Visual and auditory information were thus compatible (NoGo_compatible_). In another N = 72 NoGo trials, the word “DRÜCK” was the concurrent auditory stimulus, thus creating a conflict between the auditory word and the visual stimulus (NoGo_incompatible_), which increases demands on response inhibition. The remaining 144 NoGo trials were not accompanied by an auditory stimulus (NoGo_without_). Thus, 50% of Nogo trials were uni-modal visual and the other 50% of Nogo trials were audio-visual. Since the uni-modal NoGo condition occurred as often as the multi-modal Nogo conditions differential group effects in the uni-modal Nogo condition (NoGo_without_) cannot reflect a trivial frequency effect.

Go trials required the participants to press a response key, whenever the word “press” (German: “DRÜCK”) was presented. N = 336 Go trials were presented without any additional auditory stimulus and N = 336 trials with compatible auditory stimulus (i.e. the spoken word “DRÜCK” was presented). All participants received the instruction to respond only to visual stimuli and to ignore auditory stimuli. The higher ratio of Go trials than NoGo trials induces a pre-potent response tendency and increases demands on response inhibition processes. Trials were randomized and divided into six blocks with equal trial numbers. It was ensured that the frequency of the different Go and NoGo conditions was presented in each block. The inter-trial intervals varied between 1700 and 2100 ms. Each trial was terminated after the response. If no response was executed, the trial was terminated after 1000 ms. Go trials were rated as correct, if the response was given within 1000 ms after stimulus presentation. NoGo trials were treated as correct, if no response was executed within these 1000 ms. A standardized instruction was given and a practice run of 60 trials was executed to familiarize participants with the task. The rate of false alarms in each Nogo trial condition were calculated by counting the number of erroneous responses in a particular condition and dividing that number by the number of trials presented in each Nogo trial condition. For the accuracy on Go trials, the number of trials with correct responses were counted and divided by the number of presented Go trials. These parameters were used for the statistical analysis.

### Comprehensive behavior intervention for tics (CBIT)

The CBIT was conducted according to previously established procedures and therapy manuals^20,26^. Briefly, CBIT was administered as an individualized intervention and occasionally a parent was included in sessions. CBIT includes HRT as a competing response training, but also psychoeducation about tic disorders, tic-awareness training, relaxation training, and functional analysis. Functional analysis identifies the events and situations that influence tics and develops strategies to manage these situations. Awareness training involves the detection of premonitory urges, which helps the patient to foresee a tic and intervene early. Psychoeducation included information about the neurobiological and genetic nature of tics including information about the clinical course of the disease. During competing response training, the patient is trained to develop behavior that is physically incompatible with the tic. For that, the patients learn to make use of the premonitory urges. The CBIT intervention was conducted by VP and BB who hold a master’s degree in clinical psychology and were previously trained to reliability conduct CBIT as outlined in the manual^26^. Supervision took place on a weekly basis by VR and all treatment sessions were video-taped. CBIT was conducted for a period of 8 weeks with weekly sessions. The first session was 90 minutes, all other sessions 60 minutes long. All patients were invited to a booster session 3 months after the end of the CBIT.

### Statistical analyses

Mixed effects ANOVAs were used to analyze the behavioral data (hits, misses and FA rates, as well as corresponding RTs). The factor “group” (GTS vs. HCs) was used as between-subject factor. For the analysis of Nogo trials, the factor “condition” (NoGo_without_/NoGo_compatible_/NoGo_incompatible_) was used as within-subject factor. In all analyses, the factor “time point” (before vs. after CBIT in the GTS group) served as additional within-subject factor. The analysis of Go trials comprised the factor “condition” (i.e., Go_without_ and the Go_compatible_). Greenhouse-Geisser correction was applied and all post-hoc tests were Bonferroni-corrected. For the descriptive statistics the mean and standard error of the mean (S.E.M.) are given. In addition to classical statistics, also Bayesian statistics were carried out to quantify the evidence for the null hypothesis in the case of non-significant results in the ANOVA. This is important because the hypotheses actually depend on the lack of differences in one task condition after the HRT. To do so, we used the method by Masson^38^, in which the probability of the null hypothesis being true given the obtained data *p*(H_0_/D) can be calculated on the basis of the ANOVA results.

## Data Availability

The datasets generated during and/or analyzed during the current study are available from the corresponding author on reasonable request.
